# Multi-Level Regulatory Interactions between NF-κB and the Pluripotency Factor Lin28

**DOI:** 10.3390/cells9122710

**Published:** 2020-12-17

**Authors:** William T. Mills, Noor N. Nassar, Deepa Ravindra, Xinbei Li, Mollie K. Meffert

**Affiliations:** 1 Department of Biological Chemistry, Johns Hopkins University School of Medicine, Baltimore, MD 21205, USA; William.Mills@jhmi.edu (W.T.M.IV); nnassar3@jhu.edu (N.N.N.); dravind1@jhu.edu (D.R.); xli202@jhmi.edu (X.L.); 2 Solomon H. Snyder Department of Neuroscience, Johns Hopkins University School of Medicine, Baltimore, MD 21205, USA

**Keywords:** transcription factor, NF-κB, Lin28, let-7 microRNA, pluripotency, neurodevelopment, nervous system, RNA-binding protein

## Abstract

An appreciation for the complex interactions between the NF-κB transcription factor and the Lin28 RNA binding protein/let-7 microRNA pathways has grown substantially over the past decade. Both the NF-κB and Lin28/let-7 pathways are master regulators impacting cell survival, growth and proliferation, and an understanding of how interfaces between these pathways participate in governing pluripotency, progenitor differentiation, and neuroplastic responses remains an emerging area of research. In this review, we provide a concise summary of the respective pathways and focus on the function of signaling interactions at both the transcriptional and post-transcriptional levels. Regulatory loops capable of providing both reinforcing and extinguishing feedback have been described. We highlight convergent findings in disparate biological systems and indicate future directions for investigation.

## 1. Introduction

While using mobility shift assays to study the gene rearrangement events that lead to antibody diversity, Ranjan Sen and David Baltimore discovered a nuclear factor that bound to the κ light-chain enhancer in extracts from B cell tumors and so called it nuclear factor kappa B (NF-κB) [1]. A series of subsequent investigations showed that NF-κB activity could be induced without new protein synthesis [2], that it exists in an inhibited latent form in the cytoplasm [3], and that this retention is mediated by inhibitor of NF-κB (IκB) proteins [4]. NF-κB is now known to be expressed in essentially all cell types with a vast array of activators and functional outputs. While NF-κB has been most widely studied in the immune system, a growing body of literature has examined its role in embryonic stem cells and the premature and mature nervous system [5,6,7].

The details of NF-κB activation and function, reviewed extensively elsewhere [8,9,10], are briefly summarized here. NF-κB exists as a homo- or heterodimer composed of five potential subunits: RelA (p65), RelB, c-Rel, p50, and p52. RelA, RelB, and c-Rel are synthesized as mature proteins and contain transcription activation domains (TADs) and the p50 and p52 subunits are the N-terminal cleavage products of the precursor proteins p105 and p100, respectively. While p50 is generated constitutively, the generation of p52 is signal-induced. Under basal conditions, the latent NF-κB dimer remains in the cytoplasm bound by inhibitory IκB proteins. Members of the IκB group include IκBα, IκBβ, IκBε, the nuclear IκBs (BCL-3, IκBζ, IκBNS), and the C-terminal portions of p105 (IκBγ) and p100 (IκBδ). Each IκB contains tandem ankyrin repeats that bind NF-κB and occlude the nuclear localization sequence (NLS) in NF-κB dimers. The initial step in NF-κB activation involves stimulus-induced modification and removal of the IκB inhibitor. IκB is phosphorylated at two critical serine residues by the IκB kinase complex (IKK), which leads to subsequent polyubiquitination and degradation of IκB by the proteasome. Degradation of IκB exposes the NLS on NF-κB and allows for the dimer to stably translocate to the nucleus where it can bind cognate κB sites in the promoters and enhancers of target genes and regulate their transcription. IKK-mediated activation of NF-κB occurs through either the canonical or alternative pathway. In the canonical pathway, the IKKα/IKKβ complex phosphorylates IκBα at Ser32 and Ser36 and IκBβ at Ser19 and Ser23. The alternative ‘noncanonical’ pathway proceeds through IKKα-mediated phosphorylation of the NF-κB precursor protein, p100, at Ser176 and Ser180 leading to proteasomal removal of an IκB-resembling ankyrin repeat in p100 and the generation of p52 and its translocation to the nucleus with its main heterodimer partner, RelB.

Critical features of the NF-κB pathway include the poised dimers latent in the cytoplasm which permit rapid response to stimuli, the canonical feedback mechanism in which NF-κB-mediated IκB induction allows regulated resolution of activation, and the numerous and diverse array of NF-κB activators and NF-κB-regulated genes (online resource: http://www.bu.edu/nf-kb/gene-resources/target-genes/). Collectively, these features contribute to the prominent function of NF-κB in a plethora of biological systems, including the mature and developing nervous system. In this review, we will focus on a more recently appreciated NF-κB target gene, the Lin28 RNA binding protein, and how interactions between the NF-κB and Lin28 pathways expand the repertoire of NF-κB to encompass largely reinforcing post-transcriptional gene regulatory mechanisms.

## 2. Lin28 Paralogs

Lin28 is an RNA-binding protein that was originally discovered in *Caenorhabditis elegans* as a regulator of developmental timing and has since been shown to exhibit functional and sequence conservation in *Xenopus*, zebrafish, *Drosophila*, mouse, and humans [11,12,13,14,15]. Unlike *C. elegans* with a single *LIN28* gene, mammals have two Lin28 paralogs, possibly having arisen from a gene duplication event, which encode the Lin28a (discovered first and originally referred to as Lin-28) and Lin28b proteins [16]. Lin28a and Lin28b share extensive amino acid sequence homology and each contain multiple RNA-binding domains: a cold shock domain (CSD) and two CCHC zinc knuckle domains. Lin28a and Lin28b can each regulate translation via direct binding to mRNAs and by regulating the biogenesis of precursor microRNAs (miRNAs) containing a Lin28 recognition motif, principally the Lethal-7 (let-7) miRNA family [17]. Let-7, one of the first discovered miRNAs, was also initially discovered in *C. elegans* as a regulator of developmental timing [18], but was subsequently found to be widely distributed in bilateral animals [19].

Let-7, like most miRNAs, begins as an RNA polymerase II-dependent transcript called the primary miRNA transcript (pri-miRNA). The pri-miRNA is processed in the nucleus by the RNase III enzyme Drosha and the double-stranded RNA binding protein DGCR8 (together called the microprocessor complex) to form the 60-70-nt-long pre-miRNA. The pre-miRNA is exported from the nucleus to the cytoplasm by exportin 5 and the Ran-GTP cycle where it is processed by another RNase III enzyme, Dicer, which removes the loop of the step-loop structure to produce a 22-bp RNA duplex. One of the strands of the RNA duplex is then loaded onto an Argonaute protein (Ago) and directs Ago to the 3′ untranslated region (UTR) of target mRNAs where it prevents protein synthesis by translation inhibition and/or transcript destabilization [20]. Regulation along each step of this biogenesis pathway is used to manipulate mature levels of the potent let-7 family in governing growth and pluripotency.

Sequence conservation suggested that let-7 miRNAs might act broadly to regulate gene expression in diverse animals, and this expectation has been borne out in conserved let-7 miRNA binding sites and gene regulatory networks across animal phylogeny [19]. Following observations that mature let-7 miRNAs are differentially expressed at different developmental stages [19,21,22], and that primary let-7 transcripts and pre-let-7 miRNAs are present in both undifferentiated and differentiated human embryonic stem cells (suggesting inhibition of biogenesis at a post-Drosha step) [21,23], several labs showed that Lin28 binds directly to let-7 and inhibits its maturation [24,25,26,27,28].

Unlike *C. elegans*, mammals and most vertebrates contain multiple members of the let-7 miRNA family. In humans, the 12 let-7 family members (let-7a-1, -2, -3; let-7b; let-7c; let-7d; let-7e; let-7f-1, -2; let-7g; let-7i; miR-98) (miRBase.org) are clustered at eight different chromosomal loci [17]. Collectively, these miRNAs are classified as a family because of a shared consensus ‘seed’ sequence that functions as a critical component of base-pairing with target mRNAs. Let-7 miRNAs gradually increase during development and, collectively, are amongst the most highly expressed miRNAs in the adult brain [29]. Let-7 miRNAs have been classified as tumor suppressors due to their conserved regulation of many oncogenes, pluripotency factors, and growth-related mRNAs, and because of their concerted downregulation in cancer [30]. Elevated let-7 family miRNA levels are required for differentiation, developmental transitions, and to avoid oncogenesis, but the let-7 family of miRNAs must be suppressed to allow for translation of mRNAs necessary for pluripotency, self-renewal, regeneration, and other plastic states. To achieve this regulation of mature let-7 levels, the biogenesis pathway of the let-7 family of miRNAs is tightly controlled [20].

Both Lin28 paralogs have been shown to participate in regulating mature let-7 family miRNA abundance by inhibiting let-7 miRNA processing through recognition of a bulged GGAG-like motif present in the terminal loop of most [25,31,32], but not all [32], let-7 precursor RNAs. Recognition of this motif by the Lin28 zinc knuckle domain is accompanied by binding of the Lin28 CSD to a ‘NGAU’ motif also present in the let-7 precursor RNAs [32]. Though both Lin28a and Lin28b have been seen in the nucleus as well as the cytoplasm, Lin28b is believed to localize predominately in the nucleolus while Lin28a mainly localizes to the cytoplasm [17]. Lin28b has been best characterized to inhibit the biogenesis of let-7 miRNAs by preventing nuclear Drosha-mediated processing of the pri-let-7 transcript. Lin28a functions mainly in the cytoplasm to inhibit processing of the pre-let-7 miRNA by recruiting TUT4 (Zcchc11) which catalyzes the addition of a polyuridine tail to pre-let-7 to prevent Dicer processing and promote precursor degradation by recruiting an exonuclease [17,31,33]. Lin28 exists in a negative feedback loop with let-7 as there are conserved let-7 binding sites within the 3′ UTR of both the Lin28A and Lin28B transcripts [26]. In these reinforcing loops, a reduction in let-7 levels, for example, would be expected to further elevate translation of Lin28 transcripts. The tissue and cellular expression profiles for Lin28a and Lin28b in mammals remain somewhat enigmatic, with low or undetectable levels reported in adults for most regions, with the exception of the testes, in atlas sources for mouse and human (e.g., www.proteinatlas.org). This stands in possible discrepancy to accumulating reports of Lin28a and Lin28b presence and function in adult organisms or differentiated cells and tissues [34,35,36,37,38,39,40,41,42,43,44,45,46,47], despite clear downregulation in levels from early development. Ineffective detection is not unfamiliar amongst low abundance proteins and transcripts which may not be readily quantified or documented in databases and could be contributed to by a failure to capture signal-dependent upregulation.

As previously indicated, Lin28 can also regulate translation through direct binding to mRNAs. The reported effects of Lin28 on the translation of bound mRNAs are mixed and may depend upon the mRNA or cellular setting, as illustrated in the following examples. In mouse embryonic stem cells, Lin28a localized to the periendoplasmic reticulum area and was reported to inhibit the translation of mRNAs destined for the ER by interaction with AAGNNG, AAGNG, and less frequently UGUG motifs, leading to reduced synthesis of transmembrane proteins, ER or Golgi lumen proteins, and secretory proteins [48]. Alternatively, Lin28 binding has also been shown to enhance the translation of targeted mRNAs. In human embryonic stem cells, Lin28 was shown to preferentially associate with a subset of cellular mRNAs containing Lin28-responsive elements and Lin28 downregulation shifted these mRNAs from polysomal to nonpolysomal fractions and decreased levels of proteins encoded by the targeted mRNAs [49]. Interestingly, the authors also observed that deletion of a 35-amino-acid section in the carboxyl-terminus of Lin28 prevented its interaction with RNA helicase A and was proposed to function as a dominant-negative inhibitor to decreases target mRNA translation based on polysome shift assays [49]. Also in human embryonic stem cells, a crosslinking and immunoprecipitation approach with high-throughput sequencing (CLIP-seq) revealed that Lin28 binds roughly a quarter of all human transcripts. This study defined a GGAGA sequence Lin28-binding motif within loop structures in mRNAs, similar to the interaction site of Lin28 within let-7 precursors [50]. Gene Ontology analysis revealed that genes involved in “RNA splicing” were significantly enriched among Lin28 targets and that while Lin28 appeared to have no effect on steady state mRNA levels, its overexpression increased levels of several proteins involved in splicing regulation (e.g., *FUS/TLS*, *hnRNP F*, *TDP-43* and *TIA-1*) and resulted in broad changes to alternative splicing [50].

## 3. Lin28 and NF-κB in Pluripotency and Progenitors

Lin28a and Lin28b are known for being highly expressed in early development and in undifferentiated cells, as well as during oncogenic transformation [51]. A profound role for Lin28a in pluripotency was recognized with the demonstration that Lin28a expression could be used to reprogram human somatic cells to pluripotent stem cells when combined with three other factors: Oct4, Sox2, and Nanog [52]. More recently, it was shown that Lin28b, like Lin28a, can cooperate with Oct4, Sox2, and Nanog in reprogramming fibroblasts to pluripotency and that reactivation of both the endogenous gene loci of *LIN28A* and *LIN28B* is required to reprogram with maximal efficiency [53]. Lin28a and Lin28b expression levels also correlate well with successful conversion to pluripotency in other reprogramming settings and can function to overcome the hurdle of maturation in the successful production of both mouse and human induced pluripotent stem cells (iPSCs) [53,54,55,56]. The precise functions of discrete reprogramming factors remains an active area of investigation, but one critical role for the Lin28 RNA binding proteins in mouse and human fibroblasts appears to be in regulating metabolism through mitochondrial oxidative phosphorylation to promote pluripotency [53]. Lin28a protein has also been shown to be stabilized through direct and indirect phosphorylation downstream of activation of the mitogen-activated protein kinase (MAPK) pathway, and this can serve to couple cellular signaling to the post-transcriptional control of pluripotency through heightened Lin28a levels [41,57]. The MAPK pathway is, additionally, both regulated by and an upstream activator of IKK-mediated NF-κB signaling [58,59].

In the nervous system, both Lin28 paralogs are enriched in neuronal precursor cells (NPC) and have been shown to play overlapping functions in enhancing NPC proliferation in mice and humans, and regulating neurogenic potential during early brain development [60,61]. Lin28 also controls progenitor and neuronal cell fate during postnatal neurogenesis [62]. Additionally, it has been shown that altering expression levels of Lin28b in cultured sympathetic neuroblasts can alter proliferation and forced expression of Lin28b in embryonic mouse sympathoadrenal neuroblasts can lead to postnatal neuroblastoma formation; however, some of these effects may be let-7 independent [63]. Several reports also show that Lin28 can mediate axon regeneration in both the murine PNS and CNS [36,64].

Like Lin28, NF-κB has been shown to play crucial roles in neurogenesis and neural stem cells [65]; specifically, NF-κB appears to be more involved in promoting proliferation of progenitors rather than their survival [66,67]. In this context, NF-κB activation was found to elevate expression of genes involved in cell cycle progression and in the increased proliferation of rodent neural stem cells [68]. While NF-κB expression does not necessarily equate to activation, NF-κB was found early on to be highly expressed in murine radial glia and neuronal precursors in regions of neurogenesis, including the subventricular zone, during development and in adulthood [69]. A defect in neurogenesis, resulting from deficiency of the p50 subunit of NF-κB, was linked to a short-term memory defect in mice [67]. NF-κB activation has been reported to promote the retention of cells in the progenitor pool and to inhibit further differentiation to neurons during development in the mouse nervous system [70]. More recently, studies utilizing human embryonic stem cells investigated a discrete role for NF-κB in promoting neuronal differentiation specifically in committed NPCs [71]. In this in vitro context, the effects of NF-κB in promoting neuronal differentiation of NPCs were linked to an NF-κB-dependent metabolic gene program that enhanced oxidative phosphorylation [71]. Reprogramming of metabolism from an emphasis on glycolysis in NPCs towards mitochondrial oxidative phosphorylation in mature neurons has been shown to accompany neuronal differentiation [72]. Interestingly, Lin28a was also shown to increase glycolysis and oxidative phosphorylation by enhancing translation of several metabolic enzymes in the context of tissue repair in both mouse and human settings [73]. Further, both Lin28a and Lin28b have well-established regulatory roles in glucose metabolism and can enhance glucose uptake and insulin sensitivity through gene regulation downstream of lowered let-7 miRNAs [34,40].

There remain some apparent discrepancies, particularly concerning the role of NF-κB in pluripotency associated with cellular reprogramming. Inflammatory or aging-associated chronic NF-κB activation is reported in mouse studies to impair reprogramming towards iPSCs [74,75]. In contrast, NF-κB activation has been shown to promote maintenance of the undifferentiated state in healthy human iPSCs and in several of the above-mentioned studies in mouse neural stem cells [76]. One potential explanation could lie in different cell-intrinsic versus systemic outcomes of NF-κB activation; a systemic impact of NF-κB activation on inhibiting gonadotrophin-releasing hormone (GnRH) release is postulated to mediate the subsequent impairment in neurogenesis [74]. The regulation by NF-κB of gene programs associated with proliferation and pluripotency has also been linked in mouse and human studies to a variety of cancers, including those of the brain [77,78,79]. Constitutively activated NF-κB leads to increased proliferation of neural stem cells in the absence of growth factors and is a common feature of glioblastoma [79,80]. Other components of the NF-κB signaling pathway have been implicated in glioblastoma such as deletions of the IκBα gene in 24.2% of glioblastoma patients [81], which would release a check on NF-κB activation. Blockade of NF-κB activity, in contrast, is reported to inhibit readouts of pluripotency in cancer stem cells, such as self-renewal, migration, apoptosis, and expression of pluripotency-related genes [82].

## 4. Transcriptional-Level Interactions between NF-κB and the Lin28/let-7 Pathway

There are several direct interactions between NF-κB and the Lin28/let-7 pathway at the transcriptional level. Multiple publications have shown that NF-κB binding to a consensus site in the first intron of *LIN28B* regulates signal-induced expression of Lin28b [83,84]. In an immortalized cell line derived from human mammary epithelial cells that were engineered to allow for tamoxifen-inducible Src kinase activity, Iliopoulos et al. (2009) showed that transient induction of Src activity led to a sustained increase in NF-κB activation and a subsequent decrease in levels of several let-7 miRNA family members. The authors went on to demonstrate that this decrease in let-7 levels occurred as a result of increased Lin28b expression following NF-κB binding to a highly-conserved NF-κB motif within the first intron of the *LIN28B* gene. This interaction was part of a positive feedback loop in which increased Lin28b transcription led to a reduction in let-7 miRNAs followed by an increase in IL-6 (a target of let-7) and subsequent activation of NF-κB by IL-6. Though not stated explicitly in this paper, a reduction in let-7 miRNA levels would also be expected to act post-transcriptionally to elevate Lin28b levels as its transcript is regulated by let-7 binding to its 3′ UTR [15,18]. Functional binding of NF-κB to the first intron of the *LIN28B* gene was also reported in a human lung cancer cell line where the oncogenic Mucin 1 C-terminal subunit (MUC1-C) transmembrane protein functions together with the NF-κB p65/RelA subunit at the first intron of the *LIN28B* gene; it was also shown that this interaction could be disrupted by inhibitors of NF-κB or MUC1-C homodimerization [84]. This regulatory loop comprising NF-κB-mediated induction of Lin28b, and consequently lowered let-7 miRNAs, was also recently implicated in producing axon outgrowth and regulating axon guidance in mouse dorsal root ganglion (DRG) neurons. Following a nerve crush, preventing lowered let-7 miRNAs with a synthetic let-7 miRNA mimic was shown to negatively impact the secretion of netrin-1 (Ntn1), whose mRNA is targeted by let-7 miRNAs, as well as the NF-κB p65/RelA subunit [85].

The first intron of the *LIN28B* gene has also been implicated in the regulation of transcription from the *LIN28B* locus by another component of the NF-κB activation pathway, IKKβ. It was proposed that Lin28b is a key effector downstream of the NF-κB pathway in maintaining ‘stemness’ based on evidence that IKKβ inhibition or loss of function lowers Lin28b expression, and that IKKβ activation induces Lin28b expression through promoting direct binding of TCF7L2, a Wnt/β-catenin effector, to the first intron of the *LIN28B* gene [86]. Several publications have examined the ability of Lin28 to potentiate Wnt signaling. It was shown that doxycycline-induced intestine-specific overexpression of Lin28 produced tumors in which Wnt signaling was activated in mice and humans, as seen by nuclear/cytoplasmic translocation of β-catenin; mRNA levels of Wnt target genes were also elevated (e.g., Cd44, Sox9) [87]. Lin28a overexpression was observed to lead to an upregulation of proteins involved in Shh and Wnt signaling (e.g., Axin2, Gli2, N-Myc) while knockdown of Lin28a decreases mRNA levels of genes involved in these pathways (e.g., Gli1, Gli2, Axin2) in a human embryonal tumor with multilayer rosettes (ETMRs) cell line [88]. The mechanism of the interaction between Lin28a and Shh and Wnt signaling was shown to be that Lin28a overexpression reduces levels of let-7a miRNAs that normally target Gli1, Gli2, and Gli3 mRNAs [88]. Additionally, it has been shown that in mouse embryos, overexpression of Lin28a leads to an upregulation of several members of the Wnt signaling pathway including Dact3, Wnt5b, Rspo3, and Cdx4 [89]. The authors of this study also recognized that their Lin28a LOF and let-7 GOF mutant mouse embryos exhibited similar phenotypes to loss of Wnt3a in mutant mouse embryos observed previously [90,91]. LEF1, another Wnt/β-catenin effector, and β-catenin itself have been shown to occupy a site in the *LIN28A* promoter in various human breast cancer cell lines [92], leading to speculation that the NF-κB activation pathway might extend transcriptional regulation to both *LIN28A* and *LIN28B* through IKKβ. More recently, a transcriptional network including Wnt/β-catenin regulation of Lin28a and let-7 miRNAs was shown to control a circuit coordinating the induction and subsequent silencing of a multipotency program modulating neural crest progenitor identity in cranial neural crest [93]. A Wnt regulatory region located in the second intron of *LIN28A* was reported to drive position-dependent Lin28a expression across neural tube progenitor cells, in a spatial manner congruent with patterns of Wnt ligand expression. Mutation of this site prevented association with the LEF1 Wnt effector molecule, and Wnt/LEF1 loss of function prevented the regulation of both Lin28a and let-7 miRNAs [93]. Crosstalk between the NF-κB and Wnt signaling pathways has been critically linked with systems outside of neurodevelopment as well, including inflammation and cancer [94]. To our knowledge, direct regulation of *LIN28A* transcription by the NF-κB transcription factor has yet to be reported. However, elevation of Lin28b protein can lead to elevated *LIN28A* transcription through feedback loops, such as enhanced Myc transcription factor expression downstream of lowered let-7 miRNA levels (discussed below) [95].

In a potential negative feedback mechanism, NF-κB has also been shown to be capable of regulating the transcription of let-7 pri-miRNA transcripts. *LET7A3* and *LET7B* form a genomic miRNA cluster on human chromosome 22a13.31. NF-κB has been shown to regulate the production of primary miRNA transcripts from this locus in mouse and human cells through both overexpression of the p50 and p65/RelA subunits, as well as through a mutational analysis of two upstream NF-κB binding sites [96]; NF-κB binding to moderately conserved NF-κB motifs upstream of the *let-7a-3* gene was also reported by Iliopoulos et al. (2009) [83]. Interestingly, while NF-κB activation did elevate levels of the primary transcript for both let-7a and let-7b, the fully processed mature forms of the let-7a and let-7b miRNAs were unchanged and this was attributed to a concomitant NF-κB-mediated induction of the let-7 processing inhibitor, Lin28b [96]. Net effects of NF-κB activation on the Lin28/let-7 pathway could vary depending upon the cellular context. Importantly, however, NF-κB has not been reported to transcriptionally regulate other let-7 family members which could circumscribe the physiological impact of this negative feedback mechanism.

## 5. Post-Transcriptional-Level Interactions between NF-κB and the Lin28/let-7 Pathway

In addition to regulation at the level of transcription, a network of post-transcriptional interactions connects the NF-κB and Lin28/let-7 pathways. Members of the let-7 family have been shown to target components of the NF-κB signaling pathway, such as IKKα [97], IKKε [98], and IκBβ [99]. Let-7g was shown to bind to the 3′ UTR of IKKα transcripts and overexpression of let-7g was shown to reduce IKKα protein levels without significantly impacting mRNA levels in macrophages that were differentiated from human monocytic leukemia cells [97]. It was also shown that let-7g overexpression could lead to a reduction in mRNA levels of MEKK1, an upstream activator of IKK-mediated NF-κB signaling, which led to reduced phosphorylation of IKKα, IKKβ, IκBα, and IκBβ; reduced IκB degradation; and inhibited nuclear translocation of the RelA/p50 dimer, p52, and RelB in macrophages [97]. Let-7b and -7i were shown to target the 3′ UTR of IKKε transcripts and reduce protein levels of IKKε following overexpression in human glioblastoma cell lines [98]. Gene set enrichment analysis revealed that, following over- and under-expression of let-7e in primary human umbilical vein endothelial cells (HUVECs), transcripts found to be differentially expressed were involved in many important biological processes and pathways including NF-κB/MAPK; co-expression/correlation networks revealed IκBβ to be the most significantly regulated mRNA [99]. Overexpression of let-7e was also shown to increase mRNA levels of many pro-inflammatory cytokines and adhesion molecules regulated by NF-κB, including IL-6 (discussed further below) [99]. Let-7e was also shown to bind to the 3′ UTR of IκBβ transcripts in HUVECs and overexpression of let-7e significantly increased nuclear translocation of p65/RelA [99].

MiRWalk, an online resource for predicting miRNA binding sites (http://mirwalk.umm.uni-heidelberg.de/) [100], predicts many interactions between let-7 and the NF-κB subunits. High confidence let-7 seed binding sites are predicted within the 3′ UTRs of p100 (score = 1), RelA (Score = 0.923), RelB (score = 1), and c-Rel (score = 1) (Table 1). High confidence predictions are scores close to 1, where the score is calculated using a random-forest based approach with the TarPmiR algorithm that uses features of binding such as folding energy, seed match, and accessibility, among others [101]. Though not considered the canonical site of miRNA binding, there are also high confidence let-7 seed binding sites predicted within the coding sequence (CDS) of p105 (score = 1) (Table 1). In these post-transcriptional interactions, a decrease in let-7 miRNA levels could elevate the levels of NF-κB activating kinases (IKKα, IKKε) to increase overall induction of NF-κB-dependent gene expression, including Lin28b, in a reinforcing interaction.

The Lin28/let-7 pathway also regulates NF-κB activity through less direct modulation of NF-κB signaling molecules by the let-7 miRNAs; interactions which would either enhance or repress NF-κB activation have been reported. Though not an exhaustive list, Table 2 summarizes examples of let-7 miRNAs which bind to the 3′ UTR and regulate translation of mRNAs that encode proteins with key roles in modulating NF-κB activity. Let-7a and -7i miRNAs were shown to target the 3′ UTR of TNFAIP3 (A20), a negative regulator of NF-κB, and overexpression of let-7a and -7i led to decreased TNFAIP3/A20 protein levels, increased phosphorylation and sustained degradation of IκBα, and enhanced phosphorylation of p65/RelA following TNFα stimulation as well as increased cytokine production following SeV infection of HEK293T cells [102]. Kidney tissue samples from lupus nephritis patients were shown to exhibit elevated let-7a, -7e, -7i, and 7j miRNA levels and decreased TNFAIP3/A20 protein levels compared to controls [102]. The 3′ UTR of TNFAIP3/A20 was also shown to be targeted by let-7 and it was observed that following *Mycobacterium tuberculosis* (Mtb) infection of cultured macrophages and mice, levels of let-7f decreased while levels of TNFAIP3/A20 increased [103]. Overexpression of let-7f diminished Mtb survival and increased NF-κB activation as measured by nuclear translocation of p65/RelA [103]. In murine and human osteosarcoma models it was shown that let-7g targets the 3′ UTR of HOXB1, a suggested inhibitor of NF-κB signaling, and that inhibition of let-7g elevates levels of HOXB1 at the mRNA and protein level which coincides with a decrease in the expression of p50 and p65/RelA at the mRNA and protein level [104]. Let-7 miRNAs have also been shown to target IL-6, what could be called a feed-forward activator of NF-κB as it is both induced by NF-κB and also activates NF-κB [105,106]. Iliopoulos et al. (2009) showed that, concurrent with the previously discussed NF-κB-mediated transcriptional induction of Lin28b following tamoxifen-inducible Src-mediated transformation of an engineered mammary epithelial cell line, lowered let-7a levels could produce elevated IL-6 mRNA which is directly targeted in the 3′ UTR by let-7a. Inhibition of let-7a or overexpression of Lin28b was observed to elevate IL-6 mRNA levels, and overexpression of let-7a or inhibition of Lin28b also inhibited STAT3 phosphorylation, a downstream target of IL-6 [83]. The 3′UTR of IL-6 was also shown to be targeted by let-7c in a human bone marrow-derived mesenchymal stem cell line and overexpression of let-7c led to a decrease in IL-6 mRNA and protein levels while inhibition of let-7c produced the opposite effect [107]. Ras, a small GTPase that induces NF-κB-mediated gene expression [108,109,110,111,112], is also regulated by let-7a binding to its 3′ UTR and overexpression of let-7a in a human liver cancer cell line reduced RAS protein levels [113]. Let-7 miRNAs additionally target the 3′ UTR of κB-Ras2, an inhibitor of NF-κB signaling that binds to IκBα and prevents its IKK-mediated phosphorylation and degradation [114,115] following LPS treatment of human macrophages [116]. Let-7a was shown to be induced following LPS treatment and this induction could be blocked by inhibition of NF-κB or pre-treatment with estradiol; overexpression of let-7a also reduced levels of κB-Ras2 mRNA [116].

The Myc transcriptional regulator, a well-studied pluripotency gene, also plays a key role in upregulating the transcripts of both Lin28a and Lin28b [95,118,119,120]. The Myc transcript is, itself, post-transcriptionally repressed by let-7 miRNAs [121,122], meaning that Myc exists in a finely tuned circuit with Lin28 induction and consequent let-7 miRNA level reduction able to further induce Myc [95]. NF-κB is woven into this signaling mechanism through transcriptionally targeting both *LIN28B* and *MYC* [123,124].

## 6. Conclusions, Ongoing Questions, Future Directions

**Figure 1:**
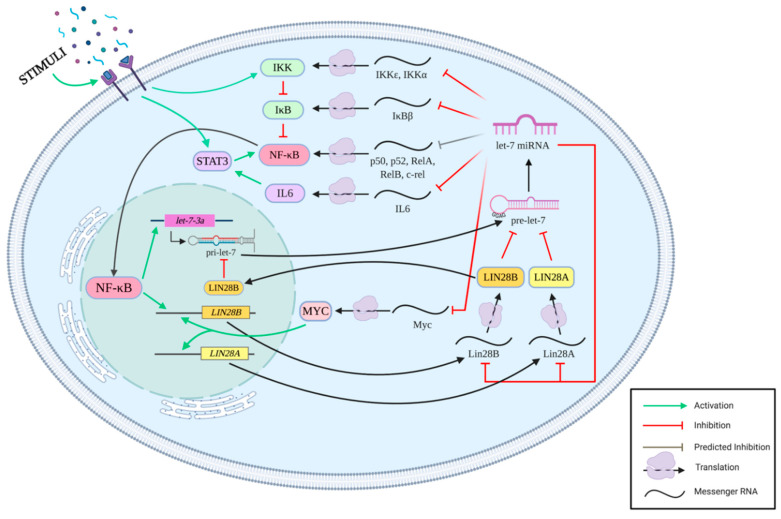
Diagram highlighting prominent pre- and post-transcriptional interactions between NF-κB and the Lin28/let-7 pathways. Core components of the NF-κB activation pathway are impacted by let-7 miRNA levels [83,97,98,99,107,121,122], and the Lin28/let-7 regulatory loops are governed by NF-κB-dependent gene expression [83,84,95,96,118,119,120]. Figure created with BioRender.com.

NF-κB has been one of the most widely studied transcription factors over the past several decades. Its roles in the immune system have been explored in-depth while its functions in the nervous system are a more recent focus of investigations. The impact of NF-κB on proliferation of neural progenitors shows similarities to the widely studied pluripotency factor Lin28 and the let-7 family of miRNAs that it regulates. There are many examples of the Lin28/let-7 pathway intersecting with NF-κB signaling at both a pre-and post-transcriptional level (Figure 1). These findings should spark an interest in better understanding how these two pathways interact and influence gene expression at the pre- and post-transcriptional level.

While the net vector of NF-κB and Lin28/let-7 pathway interactions appears most often to result in a positive feedback loop, there are several key negative regulatory interactions posited as well. In the main positive feedback loop, increased NF-κB activity leads to increased transcription of Lin28b which reduces levels of let-7 miRNAs that normally inhibit Lin28 transcripts and activators of NF-κB signaling such as IL-6. De-repression of Lin28 is expected to further reduce let-7 miRNA levels while de-repression of IL-6 would further enhance NF-κB signaling. In negative interactions, decreased let-7 miRNA levels can relieve repression of TNFAIP3/A20 and IκBβ transcripts which would be expected to repress NF-κB signaling. In future studies, insights may be gained by examining in what biological contexts negative feedback, such as transcriptional upregulation of let-7a-3 by NF-κB or the enhanced translation of TNFAIP3/A20 by lowered let-7 miRNA levels, may serve to delimit in amplitude or time, or terminate NF-κB-Lin28/let-7 pathway reinforcement. It is possible that negative interactions of this type could separate physiological from pathophysiological (e.g., oncogenesis) signaling between these potent gene regulatory pathways.

While the interactions between let-7 miRNAs and the NF-κB subunits in Table 1 are predicted bioinformatically, to our knowledge there have yet been no confirmed interactions in the literature. However, recently developed methods such as CLEAR-CLIP that allow for unambiguous determination of miRNA-target interactions could be leveraged genome-wide in cancer research to determine whether or not these predicted interactions are biologically relevant [125].

As is evident in this review, many of the experiments that have initially revealed interactions between NF-κB and the Lin28/let-7 pathway were performed in the context of cancer or transformed cell lines. As induced pluripotent stem cells (iPSCs) and new transgenic mouse lines become more readily available, it will be interesting to see whether and how these interactions occur in a stem cell context and within specific cell types of the brain and what effect they may have on development, neuronal function, and synaptic plasticity. Multiple papers have provided evidence regarding the importance of NF-κB in a variety of forms of neuronal plasticity and synapse development, however, it is only relatively recently that a link between the Lin28/let-7 pathway and neuronal plasticity has been observed [41,46,126,127]. As the interactions between NF-κB and the Lin28/let-7 pathway become more apparent, future research can be aimed at elucidating how these interactions impact the most critical features of brain development, neuroplastic function, and neural disorders.

## Figures and Tables

**Table 1:** Predicted let-7 targets among the NF-κB subunits *.

Gene	miRNA	Gene Region	Score
p105 (NFKB1)	hsa-let-7i-5p	CDS	1
	hsa-let-7e-5p		
p100 (NFKB2)	hsa-let-7e-5p	3′ UTR	1
RelA	hsa-let-7a-2-3p	3′ UTR	0.923
	hsa-let-7i-3p		
	hsa-let-7a-2-3p		
	hsa-let-7i-3p		
RelB	hsa-let-7b-3p	3′ UTR	1
	hsa-let-7a-2-3p		
	hsa-let-7a-2-3p	3′ UTR	0.923
	hsa-let-7d-3p		
	hsa-let-7b-3p		
	hsa-let-7i-5p	3′ UTR	0.872
	hsa-let-7c-3p	3′ UTR	0.846
	hsa-let-7d-5p		
	hsa-let-7e-3p		
	hsa-let-7f-5p		
c-Rel	hsa-let-7b-3p	3′ UTR	1
	hsa-let-7c-5p		
	hsa-let-7d-5p		
	hsa-let-7e-5p		
	hsa-let-7i-3p		

* Only interactions predicted to occur within the seed sequence of the miRNA have been included.

**Table 2:** Let-7 targeting of regulators of NF-κB signaling.

Gene	Activator/Inhibitor of NF-κB	Targeting miRNA ^+^	Citation
TNFAIP3 (A20)	Inhibitor	hsa-let-7a, -7e	[102]
		mmu-let-7f	[103]
HOXB1	Inhibitor	hsa-let-7g	[104,117]
IL-6		hsa-let-7a	[83]
		hsa-let-7c	[107]
Ras	Activator	hsa-let-7a	[113]
κB-Ras2	Inhibitor	hsa-let-7a	[116]
MEKK1 *	Activator	hsa-let-7g	[97]

* has-let-7g overexpression led to reduced mRNA levels of MEKK1 which is predicted to contain let-7 binding sites, but the authors did not use a luciferase reporter to confirm binding of let-7g to its 3′ UTR. ^+^ In many cases, the let-7 miRNA used for validation was just one of several let-7 family members predicted to interact with the 3′ UTR of the target transcript.
